# Genomic footprints of dryland stress adaptation in Egyptian fat-tail sheep and their divergence from East African and western Asia cohorts

**DOI:** 10.1038/s41598-017-17775-3

**Published:** 2017-12-15

**Authors:** Joram M. Mwacharo, Eui-Soo Kim, Ahmed R. Elbeltagy, Adel M. Aboul-Naga, Barbara A. Rischkowsky, Max F. Rothschild

**Affiliations:** 1 Small Ruminant Genomics Group, International Center for Agricultural Research in the Dry Areas (ICARDA), P. O. Box 5689, Addis Ababa, Ethiopia; 2 Department of Animal Science, Iowa State University, 2255 Kildee Hall, Ames, IA 50011-3150 USA; 3 Animal Production Research Institute (APRI), Agriculture Research Centre (ARC), Ministry of Agriculture, Nadi Elsaid Street, Dokki, Cairo Egypt

## Abstract

African indigenous sheep are classified as fat-tail, thin-tail and fat-rump hair sheep. The fat-tail are well adapted to dryland environments, but little is known on their genome profiles. We analyzed patterns of genomic variation by genotyping, with the Ovine SNP50K microarray, 394 individuals from five populations of fat-tail sheep from a desert environment in Egypt. Comparative inferences with other East African and western Asia fat-tail and European sheep, reveal at least two phylogeographically distinct genepools of fat-tail sheep in Africa that differ from the European genepool, suggesting separate evolutionary and breeding history. We identified 24 candidate selection sweep regions, spanning 172 potentially novel and known genes, which are enriched with genes underpinning dryland adaptation physiology. In particular, we found selection sweeps spanning genes and/or pathways associated with metabolism; response to stress, ultraviolet radiation, oxidative stress and DNA damage repair; activation of immune response; regulation of reproduction, organ function and development, body size and morphology, skin and hair pigmentation, and keratinization. Our findings provide insights on the complexity of genome architecture regarding dryland stress adaptation in the fat-tail sheep and showcase the indigenous stocks as appropriate genotypes for adaptation planning to sustain livestock production and human livelihoods, under future climates.

## Introduction

The development of high throughput genome-wide assays and associated computational tools, have made domestic livestock attractive for investigating how an organism’s genome is influenced by its production and natural environment. The existence of independent livestock populations/breeds within a species, presents a natural experimental design that can be used to study the genetic mechanisms of adaptive divergence arising from bio-physical and/or anthropological selection. For instance, genome-wide SNP and sequence data has been used to explore genetic mechanisms of adaptation to contrasting environments^1–4^ and investigate evidence for genomic selection relating to domestication, breed formation and improvement^5^ in livestock species.

Although sheep were domesticated in the Fertile Crescent, Africa is endowed with a diverse repository of the species represented by 179 breeds/populations^6^ that have been classified into three broad groups; thin-tail, fat-tail and fat-rump hair sheep. The thin-tails occur in Sudan and in the sub-humid and humid regions of West Africa. The fat-tails occur across the deserts of northern Africa, and in the highlands, semi-arid and arid environments of eastern and southern Africa. The fat-rumps are found exclusively in the semi-arid and arid zones of the Horn of Africa. The thin-tails are the most ancient in the continent and were introduced, via the Isthmus of Suez and/or the southern Sinai Peninsula, whereas the fat-tail’s arrived much later, initially via northeastern Africa (Egypt) and later via the Horn of Africa (Ethiopia)^7^. The origin of the fat-rumps remains unknown.

The genomes of African indigenous sheep have been subjected mainly to natural selection driven by tropical and sub-tropical climates, diseases and parasites. Although their productivity is much lower than that of commercial breeds under intensive production systems, indigenous sheep are often the only option available to millions of resource-poor farmers in agro-pastoral and pastoral production systems, where exotic improved genotypes under-perform under limited (quality and quantity) feed and water resources, and high ecto- and endo-parasite and disease challenges. This is evident in Egypt, a country within the Sahara desert, where sheep of the fat-tail type, are preferred by livestock keepers because of their excellent adaptation to desert-like conditions^8^. This adaptation and the historical significance of Egypt as one of the centers of dispersion of domesticates into Africa, makes Egyptian indigenous sheep of interest in understanding the genetic history of indigenous fat-tail sheep in the continent and the genomic mechanisms underlying their adaptation to dryland environments, which remain poorly investigated. Here, we generated genotype data using the Ovine SNP50K BeadChip from five populations of Egyptian indigenous sheep representative of the fat-tail hair sheep diversity found across the dry belts of Africa, the Middle East and Asia to investigate their genome profiles. Comparative genome analysis with fat-tail sheep from East Africa and western Asia provided insights on the history of the genotype in northeastern and East Africa regions, and the subsequent inclusion of European commercial breeds in selection sweep analysis, allowed us to identify unique genome profiles of fat-tail hair sheep to dryland adaptation.

## Results

### Population genetic analysis

**Figure 1:**
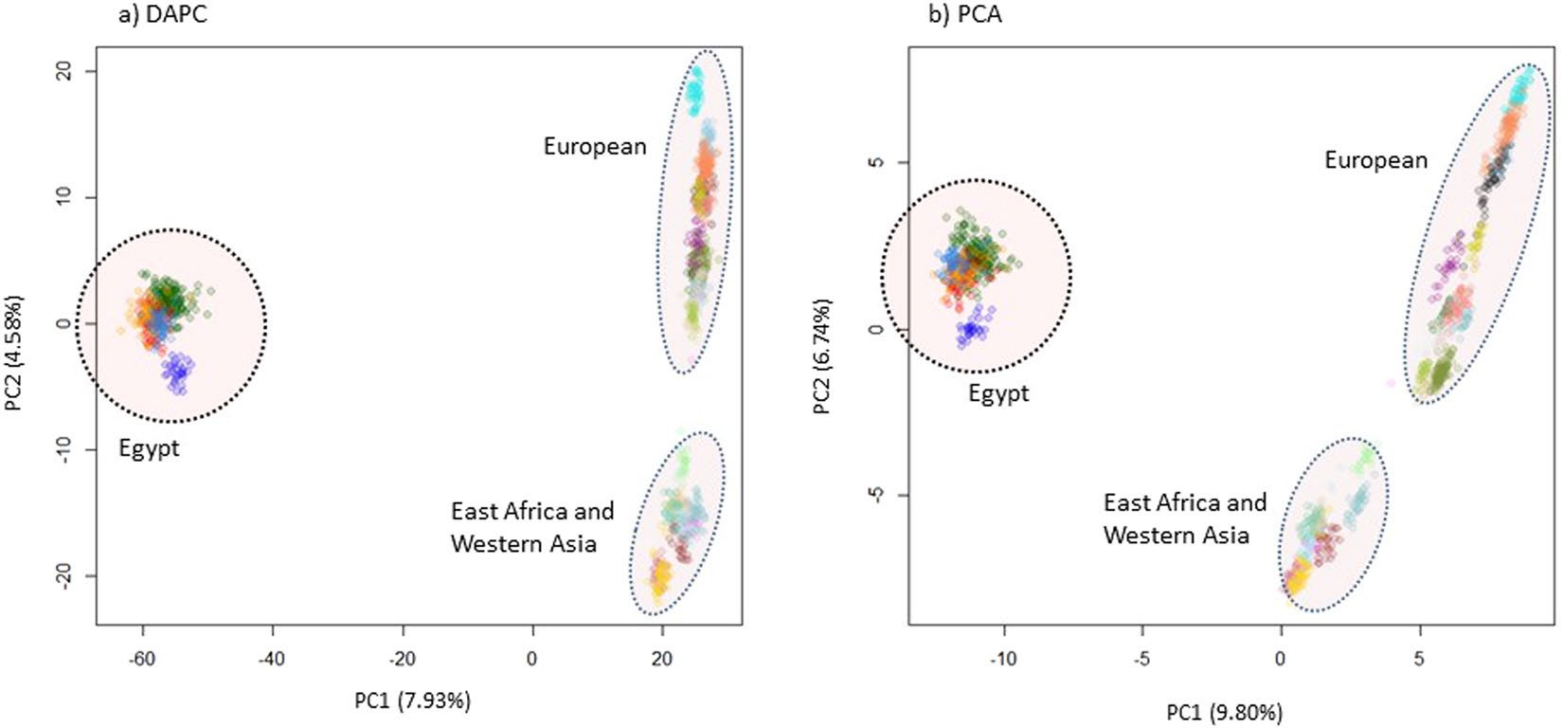
Population structure of the study populations revealed by (**a**) DAPC and (**b**) PCA analyses.

Population genetic relationships were assessed with PCA and DAPC (Fig. 1) using 5,140 SNPs that were selected to be unlinked. The first two principal components of the DAPC and PCA accounted for 7.93% and 9.80% (PC1) and 4.58% and 6.74% (PC2), respectively of the total genetic variation. The PC1 separated Egyptian and non-Egyptian populations. The PC2 separated East African and western Asia fat-tail sheep, which seem to cluster together, from the European breeds. The five Egyptian populations clustered very close together, but the non-Egyptian ones disperse along the vertical plane of the two plots. This suggests a lower level of genetic variation between the Egyptian populations but a comparatively higher one between the non-Egyptian populations. Generally, higher PCs (>2) did not result in observable genetic clusters.

### Detection of candidate selection sweep regions

**Figure 2:**
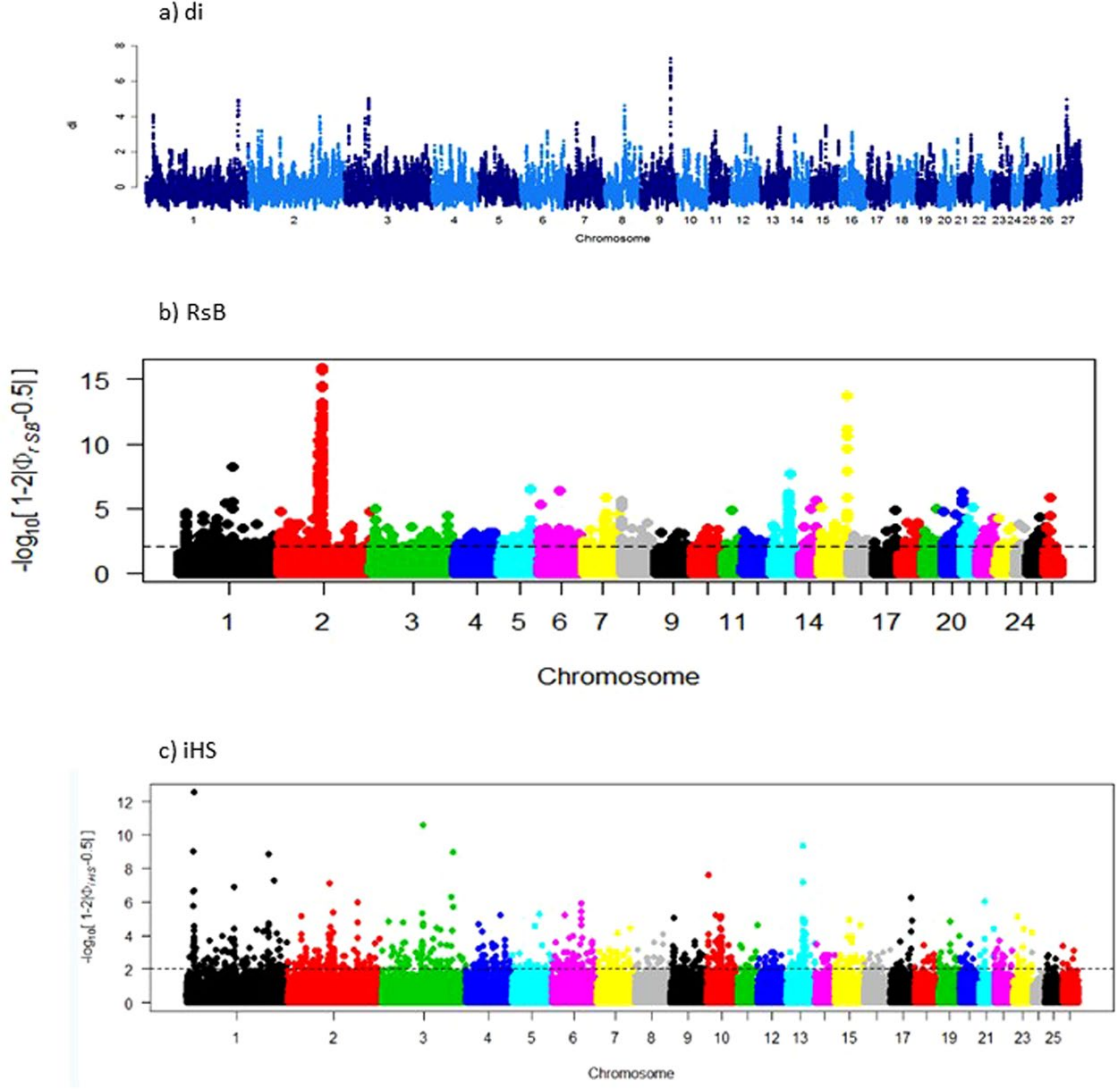
Manhattan plots for results of selection signature analysis undertaken using the (**a**) *di*, (**b**) *RsB* and (**c**) *iHS* approaches for the study populations.

Based on the results of the DAPC and PCA, SNP genotypes were used to estimate allele frequency differentiation, measured as *di*, in a pairwise comparison between the Egyptian and non-Egyptian populations. The genome wide distribution of *di* values for each SNP is shown in Fig. 2a. A total of 109 significant SNPs (*di* ≥ 4.0) defined seven candidate regions across six chromosomes (Oar1, Oar2, Oar3, Oar8, Oar9, Oar27; Fig. 2a; Supplementary Table S1a). The strongest candidate region was on Oar9 spanning 24 significant SNPs and 15 genes.

The *RsB* analysis revealed 154 significant SNPs (p*RsB* ≥ 4.0) that defined 10 candidate selection sweep regions across nine chromosomes (Oar1, Oar2, Oar5, Oar7, Oar8, Oar13, Oar15, Oar20, Oar26; Fig. 2b; Supplementary Table S1b). It identified two candidate regions as the strongest, one on Oar2 and the other on Oar15, spanning 107 and 9 significant SNPs, and, 28 and 0 genes, respectively.

The intra-population *iHS* analysis was performed for the five Egyptian populations grouped based on the PCA and DAPC. It identified 47 significant SNPs (p*iHS* ≥ 4.0) that defined 14 candidate regions across eight chromosomes (Oar1, Oar2, Oar3, Oar6, Oar10, Oar13, Oar15, Oar17; Fig. 2c; Supplementary Table S1C). Two candidate regions, that were each defined by seven significant SNPs, on Oar1 and Oar13 and spanning 46 and 17 genes, respectively were the strongest.

### Overlap between candidate selection sweep regions

The three approaches (*di*, *RsB*, *iHS*) used here to detect selection sweeps revealed 31 candidate regions across 15 chromosomes. Selection signatures were identified on Oar1, Oar2, Oar3, Oar13 and Oar15 by more than one approach. The signatures identified by the three approaches on Oar1 had an overlapping segment (*di* = 19,517,811–20,118,195 bp; *RsB* = 19,651,513–19,761,666 bp; *iHS* = 19,409,931–21,607,699 bp) (Supplementary Table S1a, S1b, S1c) spanning two genes (*TOE1, TESK2*). The signatures identified by the three approaches on Oar2, and by *di* and *iHS* on Oar3, had no overlaps. Similarly, the selection sweeps that were identified by the three approaches on Oar13 and Oar15 had no overlapping segments. Reducing the significance threshold for *di* to ≥ 3.0, resulted in overlapping segments with *iHS* on Oar13 (*di* = 57,868,286–58,232,687 bp; *iHS* = 57,771,173–58,251,812 bp) that spanned seven genes (*PCK1*, *CTCFL*, *ENSOARG00000017883*, *RAE1*, *SPO11*, *BMP7*, *ZBP1*), and Oar15 (*di* = 43,962,190–44,595,229 bp; *iHS* = 44,112,180–44,332,191 bp) that spanned three genes (*ENSOARG00000015306*, *ENSOARG00000017164*, *ENSOARG00000017173*).

### Gene content and functional annotation of the candidate regions

From the 31 candidate selection sweep regions, seven (*di* = 2, *RsB* = 3, *iHS* = 2) spanned no genes (Supplementary Table S1a, S1b, S1c) on the OARv4.0 genome assembly. Such regions have also been reported in cattle^5,9,10^. We investigated this further by checking the gene content of the seven regions against the Bovine UMD3.1 and *Capra hircus* V1 (ARS1 (GCF_001704415.1)) genome assemblies. Interestingly they spanned 83 and 18 genes, respectively on the bovine and caprine genomes, suggesting incomplete annotation of the ovine genome assembly. Genome-wide, we identified 172 genes mapping to 24 (*di* = 5, *Rsb* = 7, *iHS* = 12) candidate regions that were defined by 218 significant SNPs across 12 chromosomes (Supplementary Table S1a, S1b, S1c).

We performed functional enrichment for the 172 genes using the Enrichr web tool (See Supplementary Table S2). The rank based and combined score ranking gave similar results and revealed ten GO terms (Table 1) as the most significant (*P* ≤ 0.01). The genes were associated with diverse biological functions and some had roles in multiple functions. Relevant to this study was that majority of the functions were associated with adaptation to dryland environment stress (Supplementary Table S3a and S3b). They included response to feed stress; lipid, protein and carbohydrate metabolism; response to heat/temperature stimulus and oxidative stress; protection from ultraviolet radiation; regulation of immune response, DNA damage repair, transcription and translation, protein modification and RNA processing. Other functions included regulation of body size, growth and development; muscle structure, function and adaptation; kidney function and development; and reproduction and nervous system development and function.

**Table 1:** Overexpressed GO terms among the 172 candidate genes identified to be under selection in sheep.

Description	GO Term	P-value	Associated genes
**(a) Enrichment analysis**			
Regulation of stem cell maintenance	GO:2000036	P = 0.005938196	*TAL1*, *BMP7*
Granulocyte differentiation	GO:0030851	P = 0.005938196	L3MBTL3, TAL1
Vitamin biosynthetic process	GO:0009110	P = 0.007613258	AKR1A1, MMACHC
Microtubule cytoskeleton organization involved in mitosis	GO:1902850	P = 0.005172798	KIF3B, CHEK2
Spindle assembly involved in mitosis	GO:0090307	P = 0.003174766	KIF3B, CHEK2
Negative regulation of embryonic development	GO:0045992	P = 0.014909626	COL5A2, BMP7
Erythrocyte maturation	GO:0043249	P = 0.003790269	L3MBTL3, TAL1
Benzene containing compound metallic process	GO:0042537	P = 0.013736768	CMPK1, CYP4B1
Hair cell differentiation	GO:0035315	P = 0.005938196	ERCC3, MYO6
Protein-lipid complex assembly	GO:0065005	P = 0.008521348	BIN1, PLAGL2
**(b) KEGG Pathway**			
Pyrimidine metabolism	hsa00240	P = 0.049048713	POLR2D, CMPK1, DCK
Citrate cycle (TCA Cycle)	hsa00020	P = 0.022794916	IDH3B, PCK1
**(c) WikiPathway**			
mRNA processing	WP310	P = 0.036816137	ZBP1, ESRP1, ANKAR, GRSF1, KIAA1429, RAE1, SNRPB
TCA Cycle	WP434	P = 0.022794916	PDP1, IDH3B
Oxidation by Cytochrome P450	WP43/ WP1274	P = 0.012581695	CYP27C1, CYP4 × 1, CYP4B1
Splicing factor NOVA regulated synpatic proteins	WP1983	P = 0.042470409	KCNJ6, EPB41L2
miRNA targets in ECM and membrane receptors	WP2911	P = 0.040651645	COL3A1, COL5A2

Comparisons against the KEGG pathway and WikiPathway databases (Supplementary Table S2), revealed two and five pathways (Table 1), respectively as the most significant (*P* < 0.05). In general, the analysis of GO terms shows an over-representation of GO categories in pathways relating to stress response and which may underlie dryland stress adaptation in the fat-tail sheep (Supplementary Table S4a, S4b, S4c).

**Table 2:** Sample sizes and information on the goat populations and breeds used in the current study.

Region/Ecology	Type	Country	Breed/Population	Sample size
North Africa (Subtropical dryland)	Indigenous	Egypt	Barki	181
			Saidi	72
			Farafra	62
			Souhagi	49
			AHS	30
			Total	394
East Africa (Tropical Highland)	Indigenous	Ethiopia	Menz	34
		Kenya	Red Maasai	45
Western Asia (Subtropical)	Indigenous	Cyprus	Cyprus fat tail	30
		Iran	Afshari	37
			Moghani	34
			Qezel	35
		Turkey	Karakas	18
			Norduz	20
			Sakiz	22
			Total	196
North/Central Europe (Temperate)	Selected	Switzerland	Bundner Oberlander	21
			Swiss White Alpine	21
			Valais Black Nose	21
			Valais Red	21
		Germany	East Friesian Brown	39
			East Friesian White	9
		England	Border Leicester	48
			Dorset Horn	21
			Wiltshire	23
		Ireland	Gallway	49
			Irish Suffolk	55
		Scotland	Scottish Texel	80
		Germany	German Texel	43
		Norway	Old Norwegian Spaelsau	15
			Spael coloured	3
		Finland	Finnsheep	96
			Total	565

## Discussion

Occurring predominantly in the Afro-Asiatic drybelts, the fat-tail sheep account for approximately 25% of the global sheep population. Here, we analysed genotype data generated with the Ovine SNP50K Chip to investigate the genome profiles and the genetic basis of adaptation to dry environments in fat-tail sheep from a desert environment in Egypt. The inclusion of fat-tail sheep from East Africa and western Asia allowed us to gain insights on the history of the fat-tails in Africa. Due to their geographic proximity and ancient history of commercial and religious interactions, we hypothesized that the fat-tail sheep from Egypt and western Asia should show a close genetic relationship. However, the DAPC and PCA showed a genetic divergence between the Egyptian and western Asia and East African populations, respectively and a close genetic relationship between the latter. This confirms the results of a previous analysis with microsatellites in a large sample of African indigenous sheep that showed a clear divergence between Egypt’s fat-tail Ossimi and its East and southern African counterparts^11^. These results indicate that Egyptian and East African fat-tail sheep represent different genetic stocks, and suggest one of two possibilities; an independent introduction of at least two genepools of fat-tail sheep to Africa, or an introduction of one genetic stock that gave rise to two genepools following reproductive isolation and adaptation to different eco-climates. We favour the first suggestion which is in line with archaeological evidence which supports two separate entry points of fat-tail sheep into Africa, initially via northeast Africa (around 7500 and 7000 years ago) and later (around 5000 years ago) via the Horn of Africa^12^. The close genetic relationship between the East African and western Asia populations is difficult to explain but we suggest that it may be due to their recent common history. On the other hand, long-term reproductive isolation may explain the divergence of the Egyptian from western Asia populations. Intensive anthropological selection and/or genetic drift arising from low effective population sizes in the European breeds may explain their divergence from the African and western Asia populations.

The genetic divergence revealed by DAPC and PCA informed the grouping of populations into Egyptian and non-Egyptian ones for selection sweep analysis. The three approaches (*di*, *iHS*, *Rsb*) detected one overlapping candidate region, and two were detected with *di* and *iHS* after relaxing the stringency of *di*. A modest number of overlapping candidate regions have been reported in several studies^4,13–15^ and Bahbahani *et al*.^9^ reported none. Although coincident signatures that are detected by multiple approaches may provide strong evidence of selection^16^, their modest occurrence may be due to algorithm differences^17,18^. This may also explain why results from different studies also tend to differ. Therefore, a genomic region that has been identified by only one approach does not exclude the possibility that it could be under selection^19,20^. In total therefore, we detected 24 candidate regions, spanning 172 candidate genes, representing signatures of past and/or on-going selection in Egyptian fat-tail sheep. Unsurprisingly, the regions spanned genes that did not concern production, but rather, adaptation traits. This is because the target populations have mainly been exposed, over a long time period, to complex interacting biophysical stressors (heat, solar radiation, physical exhaustion, resource scarcity, parasites etc.) which impact fitness. This may be the reason why the spanned genes were associated with diverse physiological, molecular and cellular processes and pathways (Supplementary Table S3) emphasizing the importance of multi-functionality for adaptation to dryland environments. The large number of candidate regions and genes detected is also not surprising; similar findings have been reported for livestock species from extreme environments^4,13,15,21^. It reinforces the fact that adaptation is a complex trait that involves many biological processes and quantitative trait loci each having a small and cumulative effect on the overall expression of the phenotype.

Energy and nutrient metabolism is vital for herbivores in food scarce environments. Our candidate regions spanned several genes associated with feeding efficiency and regulation of metabolism and, GO clusters associated with energy metabolic processes (glycolysis/gluconeogenesis, TCA Cycle, insulin signaling pathway, pyruvate metabolism etc.). For instance the gene *PIK3R3*, which has not been reported before, regulates responses to changes in nutritional conditions as well as cellular metabolism and growth^22^ and through its association with 5′ AMP-activated protein kinase (AMPK), it serves as a metabolic master switch in response to alterations in cellular energy levels^23^. Indeed, sheep under heat stress decrease dry matter intake and rumination time by up to 76%^24^ which is related to eating efficiency and metabolic processes^25^. In the drylands most breeds tend to have small body sizes as an adaptation strategy to scarce and poor quality forages and for thermoregulation^25^. This may explain the occurrence, in candidate regions, of genes such as *BMP7*, *MSTN* (*GDF8*) and *STIL* which regulate adult and embryonic size, growth and development^22,26,27^. *BMP7* also plays a crucial role in renal function and development^28,29^. Renal vasodilation, transmembrane transport, water-salt metabolism, bicarbonate absorption, water retention and reabsorption are key functions of the renal cortex and central to desert environment adaptation.

Prolonged exposure to intense solar and ultraviolet radiation, which are key abiotic stressors in arid environments, can result in ophthalmic and skin conditions. Genes controlling pigmentation of coat and skin^30^ and eyelids^31^ and photoreception and visual protection^32^ have been identified and reported to offer protection against solar and UV radiation. None of these reported genes however, occurred in the candidate regions although our study populations had pigmented skins and coats and some such as Barki, Farafra and Souhagi had pigmented eyelids^8^. Instead, we observed *ERCC3* a major nucleotide excision repair (NER) protein. In humans, mutations in *ERCC3* result in skin disorders, such as *xeroderma pigmentosum*, *cockayne syndrome* and *trichothiodystrophy*, which result in sensitivity to UV radiation and oxidative stress^33–35^. Since NER modulates melanocyte stem cell attrition and development of non-pigmented hair^36^, *ERCC3* may be involved in maintaining and regulating the fate and behaviour of melanocyte stem cells and mature melanocytes, and thus the production of melanin which is responsible for skin, hair and eye colour. Another gene was *TGM3* which is widely expressed in skin cells, specifically keratinocytes and corneocytes. During keratinocyte differentiation, *TGM3* crosslinks structural proteins and lipids in the formation of cornified cell envelope which provides the barrier function of epidermis against harmful environmental stimuli such as UV radiation^37–39^.

Our candidate regions spanned several genes (*PMS1*, *SPO11*, *RAD54L*, *MUTYH*, *CHEK2*, *POLR2D*, *CMPK1*) that maintain cellular functions and DNA repair. For instance, *MUTYH* repairs 8-oxo-G (a mutagenic product of oxidative DNA damage) in the nucleus and mitochondria, via the base excision repair pathway^40,41^. *PMS1* which belongs to the mutL/hexB family of DNA mismatch repair proteins, also forms heterodimers with *MLH1*, a DNA mismatch repair protein^42^. Knockdown of *CMPK1* was observed to delay DNA repair during recovery from UV damage, suggesting it contributes to the efficiency of the DNA repair process^43^. This finding could be associated with the fact that long term exposure to acute and chronic heat stress enhances the production of free radicals which can result in DNA damage and induce oxidative stress leading to mitochondria damage^44–46^, apoptosis and necrosis^47,48^. Therefore the DNA repair system serves to preserve genomic integrity under excessive exposure to UV radiation in dryland environments.

The initiation of stress response involves the activation of the neuroendocrine system to trigger physiological and/or behavioural responses. We found several genes involved in the development of the nervous system and eliciting response to stress, indicating the importance of the neuroendocrine system in activating stress response in the candidate regions. One of the genes, *DMBX1* is involved in brain and sensory organ development^49,50^ while *TP53INP1* is a key cell stress response protein with antioxidant function^51,52^. Through its interaction with the ERK1 and p38 mitogen-activated protein kinases, *MKNK1* may play a role in responding to environmental stress and control cytokine production and delayed apoptosis^53,54^. *PLAGL2* is also an oxidative stress responding regulator^55^. The activation of the neuroendocrine system and initiation of stress response results however, in the chronic production of glucocorticoids and catecholamines, which can dysregulate immune functions^56,57^, and corticosteroids which possess potent immunosuppressive properties in lymphocytes^58^. This appears to be counterintuitive. However, we observed several genes such as *ZBP1*, *PRDX1*, *MAST2* and *LURAP* in the candidate regions that enhance immune functions. Indeed some genes such as *MAST2* and *PRDX1* have dual roles. By controlling the activities of TRAF6 and NF-kB; *MAST2* regulates immune response and acts as the first responder to harmful cellular stimuli such as stress, free radicals and UV radiation^59,60^. While *PRDX1* may play an antioxidant protective role in cells, it also contributes to antiviral activity of CD8(+) T-cells^61,62^. The *ZBP1* activates innate immune response by binding foreign DNA, enhances DNA-mediated induction of type I interferons and other genes that activate innate immune responses, as well as, signaling mechanisms underlying DNA-associated antimicrobial immunity and autoimmune disorders^63,64^. *LURAP1* is an activator of the canonical NF-kB pathway and drives the production of proinflammatory cytokines^65^. Taken together, these findings suggest that genes evoking cellular stress and immune responses have been the subject of selection in the course of adaptive evolution to dryland environments.

Thermal stress compromises fertility through a direct effect of hyperthermia on the reproduction axis or through the indirect effect of thermal stress on feed intake to reduce metabolic heat production, leading to changes in energy balance and nutrient availability. Four genes (*CTCFL*, *MAST2*, *TESK2*, *SPO11*) associated with male reproduction physiology were detected in the candidate regions. *CTCFL* is a testis-specific DNA binding protein that forms methylation-sensitive insulators which regulate X-chromosome inactivation, nuclear architecture and transcription^66,67^. *MAST2* ^68^ and *TESK2* ^69^ play an important role in spermatid maturation during spermiogenesis and spermatogenesis, respectively. In mouse, knockout of *SPO11* led to meiotic arrest of spermatocytes at zygotene^70,71^ resulting in sterility in *SPO11* ^−/−^ homozygous male mice and atrophied testes. Since thermal stress compromises fertility through a direct effect of hyperthermia on the reproductive axis or indirectly through the effects of thermal stress on feed intake to reduce metabolic heat production resulting in changes in energy balance and nutrient availability, our result suggests that reproductive success is a key determinant of adaptive fitness in the fat-tail sheep.

The observation that some of the genes spanned by the candidate regions are enriched for the GO term “response to hypoxia (GO:0001666; GO:0071456)” and “HIF-1 (hypoxia inducible factor-1) signaling pathway (hsa04066)” are novel findings of our study. The HIF-1 pathway and response to hypoxia plays an important role in cellular response to systemic oxygen levels and has been associated, so far, with adaptation to high altitude environments^1–3^. We suggest that this finding could be related to physical exhaustion arising from long-term trekking of long distances in search of feed and water which results in hypoxia-like conditions and oxygen debt in skeletal muscles. In addition two candidate genes (*MSTN/GDF8*, *BIN1*) occurred in the candidate regions under selection. *MSTN* tightly regulates skeletal muscle homeostasis and promotes the survival of muscle syncitia which make up type I (oxidative/slow) or type II (glycolytic/fast) muscle fibers^72^. Type I fibers are fatigue-resistant and rich in mitochondria and utilize oxidative metabolism to provide a stable and long-lasting supply of ATP^73^. They have been observed in skeletal muscles of endurance athletes and their activation entrains complex pathways that enhance physical^73^ and racing performance^74^. Isoforms of *BIN1*, are important in the formation of transverse tubules which play a role in skeletal and cardiac muscle contractility and relation^75,76^. The selection of *MSTN*, *BIN1* and HIF-1 associated genes could have arisen as an adaptive response to endure physical exhaustion arising from long-term long distance walking.

In this study, we generated a catalogue of genetic variants in Egyptian fat-tail sheep. Although the studied populations are only a subset of the fat-tail sheep found in Africa, they illustrate that at least two distinct and phylogeographically structured autosomal gene pools define the genotype in the continent. Genome-wide differentiation and LD based scans of selection sweeps identified several candidate genomic regions under selection that spanned several novel and reported genes with key adaptive physiological functions. These genes especially those associated with pigmentation, muscle function and the HIF pathway would need further investigation and validation using full genome sequences and expression studies in sheep and model species and further test hypotheses arising from our study.

## Material and Methods

### Animals

The animals used in this study are owned by farmers. Prior to sampling, the objectives of the study were explained to them in local languages so that they could make an informed decision with regard to providing consent to sample their animals. Blood sampling was performed by a licensed veterinarian following the guidelines of the General Organization for Veterinary Service (GOVS), Egypt.

### Sampling, SNP genotyping and data quality control

Venous blood was collected into EDTA vacutainer tubes from 394 individuals from five fat-tail sheep populations (Barki, Saidi, Farafra, Souhagi, AHS) in Egypt (Table 2). The phenotypic characteristics, and socio-economic and cultural significance of the populations were described by Galal *et al*.^8^. The animals were sampled at random from farmers’ flocks where they are managed under transhumant grazing system, and veterinary care and anthropic selection is modest or not practised. The blood samples were transferred onto Whatman FTA^TM^ Classic cards (GE Healthcare UK Ltd) for storage. Genotyping was performed on FTA^TM^ spotted blood at GeneSeek Inc (http://www.neogen.com/Genomics/) on the Illumina Ovine SNP50K BeadChip. Similar genotype data from 565 individuals from 15 breeds of European sheep, 196 individuals from seven populations of western Asia fat-tail sheep and 79 individuals from two populations of fat-tail sheep from East Africa (Table 2) were obtained from the Ovine HapMap project (http://www.sheep-hapmap.org). The European breeds were used to represent genotypes from a contrasting temperate environment. The East African populations represented fat-tail sheep from a different geographic region within Africa and the western Asia ones represented fat-tails from a region close to the centre of domestication.

PLINK 1.07^77^ was used to perform data quality control and processing. A SNP was excluded from the analysis if it failed the following quality criteria; a SNP call rate greater than 95%, SNP genotyping success rate of greater than 90%, a per-SNP minor allele frequency of less than 0.03 and the presence of mapped autosomal loci. Overall, 1,234 individuals genotyped for at least 95% of the SNPs and 51,407 SNPs that passed quality thresholds were available for analysis.

### Population analyses

We performed the principal component analysis (PCA) using the *adegenet* 1.4–2 package^78^ for R^79^ to investigate genetic relationships between individuals and populations. To minimize possible confounding effects of linkage disequilibrium on the underlying pattern of population genetic relationship and structure, one in every ten SNPs was sampled and used in PCA. This dataset was also used to perform the discriminant analysis of principal components (DAPC) with *adegenet* 1.4–2 package in R, to assess replication of the population clusters and relationships between individuals and populations. The PCA aims to reduce the total variance in the dataset and identifies the principal components (PCs) that represent population structures based on genetic correlations between individuals. The DAPC reveals genetic differences between groups, as best as possible, while minimizing the variation within the groups.

### Signatures of selection based on genetic differentiation

We computed locus-specific divergence in allele frequencies between groups of populations revealed by DAPC and PCA, using the *di* statistic^80^. The *di* is a function of unbiased estimates of pairwise values of *F*_ST_ calculated for each SNP between one or a group of population(s) against the other(s). For each of the 51,407 SNPs that passed quality control, the expected value and standard deviation of *F*_ST_ were calculated. The *F*_ST_ values were then averaged across SNPs contained in non-overlapping sliding windows of 20 SNPs. The regions under selection were defined if at least two non-overlapping windows passed the distribution threshold, taking the windows with the highest (top 1%) average values of *di* as the candidate regions^80^.

### Signatures of selection based on linkage disequilibrium (LD)

LD based selection sweeps were identified using the *iHS* ^17^ and *RsB* ^18^ approaches. These approaches are based on the extended haplotype homozygosity (EHH) estimates of LD. The *iHS* detects regions with high levels of unexpected EHH within populations relative to neutral expectations and *RsB* detects the same but between populations. The analyses were performed using the rehh package^81^ in R. SNPs under selection were identified by transforming *iHS* and *RsB* values into p*iHS* (*piHS* = −log_10_[1 − 2(Φ_(*iHS*)_ − 0.5)] and p*RsB* (*pRsB* = −log_10_[1 − 2(Φ_(*RsB*)_ − 0.5)], where (Φ_(*x*)_) represents the Gaussian cumulative distribution function. Assuming the values of *iHS* and *RsB* are normally distributed under neutrality, the p*iHS* and p*RsB* can be interpreted as −log_10_(*P-value*).

For *iHS*, the intra-population Integrated Haplotype Score (*iHS score*) was computed for the five Egyptian populations as a group. For each SNP, we calculated the natural logarithm of the ratio between the integrated EHH for the ancient (*iHH*_*A*_) and derived (*iHH*_*D*_) alleles. We inferred the allelic states of each SNP using two approaches; (i) the SNP with the highest frequency was taken to be the ancestral allele; and (ii) the states were assigned at random following 100 permutations to ensure consistency. The *RsB* scores were computed between the Egyptian and non-Egyptian groups of populations. The integrated EHHS (site-specific EHH) score for each SNP and group of populations (*iES*) were calculated, and the *RsB* statistic between the two groups calculated as the natural logarithm of the ratio between *iES*_Egyptian_ and *iES*_non-Egyptian_.

Haplotypes for *iHS* and *RsB* were constructed by phasing the genotyped SNPs using Beagle^82^. Haplotype frequencies were then calculated using 20 SNP sliding windows that overlapped by five SNPs. The SNPs having p*iHS* and p*RsB* (log_10_(*P*-value)) values ≥ 4.0 (*P*-value < 0.0001), were considered significant. Candidate regions were defined if the values for at least two adjacent SNPs were significant. In cases where multiple SNPs were significant, a distance of 0.5 Kb up- and down-stream of the extreme significant SNPs was used to define the candidate regions.

### Functional annotation of candidate regions and genes

The candidate regions were annotated and the associated genes retrieved from the Ensembl Genome Browser 87 using the Sheep Genome Assembly OARv4.0 (GCF_000298735.2). Functional enrichment was performed with Enrichr^83^ against terms from the Gene Ontology (GO), KEGG (www.kanehisa.jp/) and WikiPathways (www.WikiPathways.org/index.php/WikiPathways) databases which annotates groups of genes with dedicated terms. To rank the GO terms that are relevant to the candidate genes, we assessed the significance of overlap between the input gene list and the gene sets in each library using the *z*-score test statistic of the deviation from the expected rank by the Fisher exact test (rank based ranking). This test outperforms the Fisher exact test and is comparable to the combined score ranking test^83^. The NCBI Pubmed (http://www.ncbi.nlm.nih.gov/pubmed/), GeneCards® (http://www.genecards.org/) and UniProt (http://www.uniprot.org/) databases were also used to determine the gene functions.

### Data availability

The data generated in this study is available upon request from Eui-Soo Kim (euisoo.kim@recombinetics.com).

## Electronic supplementary material

Supplementary Information

Supplementary Table S2
