# Supplementary material for: Genomic footprints of dryland stress adaptation in Egyptian fat-tail sheep and their divergence from East African and western Asia cohorts
Source: Sci Rep. 2017 Dec 15;7:17647. doi: 10.1038/s41598-017-17775-3 (PMC5732286; doi:10.1038/s41598-017-17775-3)
Supplement: Supplementary file 1 — Supplementary Information [file 41598_2017_17775_MOESM1_ESM.pdf]

**Supplementary Information/Materials**

# Genomic footprints of dryland stress adaptation in Egyptian fat-tail sheep and their divergence from East African and western Asia cohorts

Joram M. Mwacharo[1], Eui-Soo Kim [2], Ahmed R. Elbeltagy[3], Adel M. Aboul-Naga[3], Barbara A. Rischkowsky[1], Max F. Rothschild[2]

*Correspondence: Joram M. Mwacharo
Email: j.mwacharo@cgiar.org

**Supplementary Table S1. Potential candidate selection sweep regions and their genes determined from genome wide a) *di,* b) *Rsb and* c) *iHS* analysis of Egyptian indigenous goats**

**a) di analysis**

| Chromosome | From | To | No Significant SNPs | Gene(s) mapping to the region |
|---|---|---|---|---|
| 1 | 19,517,811 | 20,118,195 | 14 | 14 genes (ZSWIM5, HPDL, MUTYH, TOE1, TESK2, MMACHC, ENSOARG00000001661, PRDX1, AKR1A1, ENSOARG00000001820, CCDC17, ENSOARG00000002014, TMEM69, IPP) |
| | 267,929,090 | 268,796,349 | 16 | 4 genes (DYRK1A, KCNJ6, KCNJ15, ERG) |
| 2 | 195,458,281 | 195,852,924 | 10 | 1 gene (ACAA2) |
| 3 | 66,704,162 | 67,373,893 | 16 | No genes |
| 8 | 55,385,947 | 56,203,055 | 16 | 4 genes (L3MBTL3, SAMD3, TMEM200A, EPB41L2) |
| 9 | 81,558,829 | 82,999,383 | 24 | 15 genes (C8orf37, PLEKHF2, NDUFAF6, TP53INP1, CCNE2, INTS8, DPY19L4, ESRP1, KIAA1429, ENSOARG00000007599, ENSOARG00000007691, GEM, CDH17, ENSOARG00000008160, PDP1) |
| 27 | 58,281,285 | 59,578,440 | 13 | No genes |

**b) *Rsb analysis***

| Chromosome | From | To | No significant SNPs | Gene(s) mapping to the region |
|---|---|---|---|---|
| 1 | 19,651,513 | 19,761,666 | 2 | 2 genes (TOE1, TESK2) |
| 2 | 116,127,777 | 127,499,743 | 107 | 28 genes (UGGT1, SAP130, ENSOARG00000009427, AMMECR1L, POLR2D, LIMS2, MYO7B, IWS1, MAP3K2, ERCC3, CYP27C1, BIN1, ENSOARG00000016201, NAB1, MFSD6, ENSOARG00000016259, HIBCH, C2orf88, MSTN, ORMDL1, OSGEPL1, ANKAR, ASNSD1, SLC40A1, WDR75, COL5A2, PMS1, COL3A1 |
| 5 | 90,749,227 | 90,799,901 | 2 | 1 gene (FAM172A) |
| 7 | 66,814,857 | 67,014,339 | 3 | 1 gene (SLC35F4) |
| 8 | 2,376,421 | 2,791,283 | 7 | 3 genes (SENP6, MYO6, IMPG1) |
| 13 | 51,492,534 | 52,712,405 | 10 | 18 genes (PTPRA, VPS16, PCED1A, TMEM239, ENSOARG00000005534, CPXM1, EBF4, IDH3B, NOP56, TMC2, SNRPB, TGM6, TGM3, STK35, PDYN, ENSOARG00000007425, ENSOARG00000007600) |
| | 61,009,894 | 61,210,720 | 3 | 4 genes (POFUT1, KIF3B, ASXL1, ENSOARG00000003880)<br>2 adjacent genes (TM9SF4, PLAGL2) |
| 15 | 82,542,189 | 84,764,911 | 9 | No genes |
| 20 | 55,211,137 | 55,393,724 | 4 | No genes |
| 26 | 20,935,998 | 20,986,055 | 2 | No genes |

**c. *iHS* analysis**

| Chromosome | From | To | No significant SNPs | Gene(s) mapping to the region |
|---|---|---|---|---|
| 1 | 19409931 | 21607699 | 7 | 46 genes (EIF2B3, HECTD3, ZSWIM5, UROD, MUTYH, TOE1, TESK2, HPDL, ENSOARG00000001661, MMACHC, PRDX1, AKR1A1, ENSOARG00000001820, CCDC17, ENSOARG00000002014, TMEM69, IPP, MAST2, PIK3R3, TSPAN1, POMGNT1, LURAP1, RAD54L, LRRC41, ENSOARG00000002308, ENSOARG00000002323, NSUN4, FAAH, ENSOARG00000002492, DMBX1, KNCN, MKNK1, MOB3C, ENSOARG00000002611, TEX38, ENSOARG00000002745, CYP4B1, ENSOARG00000003003, ENSOARG00000003091, ENSOARG00000003175, ENSOARG00000003294, CYP4X1, PDZK1IP1, TAL1, STIL)<br>1 adjacent (CMPK1) |
| | 240316879 | 240919225 | 5 | 2 genes (PLSCR4, PLOD2) |
| 2 | 37064032 | 37153924 | 2 | 2 genes (UBAP1, ENSOARG00000008077)<br>1 GENE adjacent (KIF24) |

| | | | | |
|---|---|---|---|---|
| | 122878732 | 122948722 | 2 | No genes |
| 3 | 116239387 | 116527734 | 2 | 2 genes (OTOGL, PTPRQ) |
| | 203023428 | 203054874 | 2 | 1 gene (ETV6) |
| 6 | 85739817 | 86462202 | 4 | 4 gene (RUFY3, GRSF1, MOB1B, DCK) |
| 10 | 40036146 | 40150757 | 2 | 1 gene (ENSOARG00000017107) |
| | 42221999 | 42925868 | 3 | No genes |
| 13 | 51492534 | 52630089 | 7 | 17 genes (ENSOARG00000004656, PTPRA, VPS16, PCED1A, ENSOARG00000005534, TMEM239, CPXM1, EBF4, IDH3B, NOP56, TMC2, SNRPB, TGM6, TGM3, STK35, PDYN, ENSOARG00000007425) |
| | 57771173 | 58251812 | 3 | 8 genes (PMEPA1, ZBP1, PCK1, CTCFL, ENSOARG00000017883, RAE1, SPO11, BMP7) |
| | 61009894 | 61093344 | 2 | 3 genes (PLAGL2, POFUT1, KIF3B) |
| 15 | 44112160 | 44332191 | 3 | 3 genes (ENSOARG00000015306, ENSOARG00000017164, ENSOARG00000017173) |
| 17 | 67141764 | 67750997 | 3 | 5 genes (TTC28, CHEK2, HSCB, CCDC117, ENSOARG00000002962) |

Supplementary Table S3. Categories of functions and GO terms and their encoding genes associated with adaptation to dryland environment stress

S3a. Direct response functions

| Category | Function | Gene(s) |
|---|---|---|
| Feed and nutrient stress response | Metabolic adaptations to nutrient levels/Nutrient metabolic process | BMP7, TSPAN1, AKR1A1, MMACHC |
| | Carbohydrate metabolic processes | EIF2B3, CMPK1, DCK, PCK1, AKR1A1, RAE1 |
| | Protein metabolic processes | CHEK2, HPDL, ASNSD1, HIBCH |
| | Lipid metabolic processes | ACAA2, PIK3R3, PDP1, PIK3R3, FAAH, ACAA2, ASXL1 |
| | Adult feeding behaviour | DMBX1 |
| | Salivary gland morphogenesis/Digestive tract development/Odontogenesis | BMP7, COL3A1 |
| Temperature/heat response | Cellular response to heat | EIF2B3, MSTN, TP53INP1, POLR2D |
| | Skin development, keratinization, hair cell differentiation, hair follicle morphogenesis, hair molting cycle | NAB1, COL3A1, COL5A2, ERCC3, MYO6, TGM3, MYO6 |
| | Response to gamma/UV radiation | ERCC3, TP53INP1, CHEK2, RAD54L, COL3A1, RAD54L |
| | Retina homeostasis/response to light stimulus | PRDX1, ERCC3, TP53INP1 |
| | Visual perception | IMPG1 |
| Water stress/regulation | Kidney function and development | BMP7 |
| | Response to osmotic and salt stress | MKNK1 |
| Abiotic factors/Oxidative stress | Cellular response to abiotic/external stimuli | MAP3K2,TP53INP1, MYO6, TMC2 |
| | Cellular response to oxidative stress/reactive oxygen species | PRDX1, TP53INP1, ERCC3, BMP7, MAP3K2, DYRK1A |
| | Regulation of translation in response to stress | EIF2B3 |
| | Regulation of gene expression by epigenetic or genetic imprinting | CTCFL |
| Energy biosynthesis and metabolism | Gluconeogenesis | PCK1 |
| | Mitochondrial organization and function | ACAA2, KIF3B, ERCC3, KIF24, PMS1, NDUFAF6 |
| | GTP metabolic/catabolic process | GEM, EIF2B3, BIN1 |
| | TCA cycle | IDH3B |
| | Cellular metabolism process | MMACHC, AKR1A1, BIN1, PLAGL2 |
| | Energy (ATP) metabolic processes | PDP1, ERCC3, KIF3B, KIF24, PMS1, IDH3B |
| Enhanced immune functions | Cellular response to biotic stimulus | PLSCR4 |
| | Immune response | ZBP1, KIF3B, COL3A1, VPS16, L3MBTL3, TAL1, PLSCR4, PRDX1, MAST2, LURAP1, RAE1, SCL40A1 |

S3b. Indirect response functions

| Category | Function | Gene(s) |
|---|---|---|
| Organ function and development | Body size, growth and development, skeletal system development, tissue morphogenesis | COL3A1, PRDX1, COL5A2, BMP7, DMBX1, STIL, MSTN, ASXL1, GRSF1 |
| | Regulation of cell morphogenesis involved in differentiation | RUFY3 |
| | Multicellular organismal development and growth | ERG, EBF4, STIL, DMBX1, MSTN |
| | Developmental maturation | L3MBTL3, TAL1 |
| Muscle structure, development, function and adaptation | Muscle cell differentiation | BIN1 |
| | Muscle function, development, regeneration, adaptation | MSTN |
| | Somitogenesis | POFUT1 |
| Male and female reproduction | Gamete regeneration/development | SPO11, TESK2, CMPK1, MSTN |
| | Ovulation cycle process | MSTN, SPO11, CMPK1 |
| | Regulation of reproductive process | BMP7 |
| | Cellular responses involved in reproduction in multicellular organisms | SPO11, MAST2 |
| | Response to oestrogen | MSTN, BMP7 |
| | DNA methylation involved in gamete generation | CTCFL |
| | Embryonic development/morphogenesis | STIL, TAL1, PLAGL2, MYO6, GRSF1, BMP7, COL5A2, POFUT1 |
| Detection of external environment | Central and peripheral nervous system development and function | DMBX1, BIN1, BMP7, TAL1, POFUT1, DYRK1A, NAB1, RUFY3, ERCC3, MYO6, COL3A1, PTPRA, COL5A2, STIL, EIF2B3, KCNJ6, KCNJ15, PDYN |
| Control of internal environment | Cellular ion homeostasis | SLC40A1 |
| | Regulation of homeostatic process | TAL1 |
| | Anatomical structure/tissue homeostasis | PRDX1 |

Supplementary Table S4. Pathways associated with stress response as deduced from GO annotation analysis (S4a), the KEGG pathway S4b) and WikiPathways (S4c) databases

S4a. Pathways associated with stress response from GO analysis

| **Pathway** | **GO term** |
|---|---|
| Transforming growth factor beta receptor signaling pathway | GO:0007179/GO:0030512/GO:0017015/ |
| Peroxisome proliferator activated receptor (PPAR) signaling pathway | GO:0035358 |
| Type I interferon-mediated signaling pathway | GO:0060340/GO:0060338/GO:0032481/ |
| Pathway-restricted SMAD protein phosphorylation | GO:0060393/94/95/GO:0010862 |
| Insulin receptor signaling pathway | GO:0008286 |
| Integrin-mediated signaling pathway | GO:0007229/GO:2001044 |
| Apoptotic signaling pathway | GO:0097190/91/92/93/GO:0008630/GO:2001242/1233/1234/1235/1244/GO:1901028 |
| Ubiquitin-dependent protein catabolic process via the multi-vesicular body sorting pathway | GO:0006511/GO:0043162 |
| Cytokine-mediated signaling pathway | GO:0001961/GO:0019221/GO:0001959 |
| Notch signaling pathway | GO:0007219/GO:0008593/GO:0045746 |
| Transmembrane receptor protein serine/threonine kinase signaling pathway | GO:0007178/GO:0090100/GO:0090101/GO:0090092/ |
| Retinoic acid receptor signaling pathway | GO:0048385/GO:0048387 |
| Androgen receptor signaling pathway | GO:0030521 |
| Hormone-mediated signaling pathway | GO:0009755 |
| Steroid hormone mediated signaling pathway | GO:0043401/GO:0030518 |
| Intracellular steroid hormone receptor signaling pathway | GO:0030518 |
| BMP signaling pathway | GO:0030509 |
| Smoothened signaling pathway | GO:0007224 |
| Fibroblast growth factor receptor signaling pathway | GO:0008543 |
| Intracellular receptor signaling pathway | GO:0030522 |
| Neuropeptide signaling pathway | GO:0007218 |
| Stress-activated MAPK cascade | GO:0032872/GO:0043410/GO:0032874/GO:0000165/GO:0043409 |
| JNK cascade | GO:0046328/GO:0046330 |
| Ras protein signal transduction | GO:0046578/GO:0046579 |
| Stress-activated protein kinase signaling cascade | GO0070302/GO:0070304 |

S4b. Pathways associated with stress response from KEGG Pathways database

| **Pathway** | **GO term** |
|---|---|
| Insulin signaling | hsa04910 |
| p53 signaling | hsa04115 |
| Estrogen signaling | hsa04915 |
| HIF-1 signaling | hsa04066 |
| AMPK signaling | hsa04152 |
| FoxO signaling | hsa04068 |
| Oxytocin signaling | hsa04921 |
| Hippo signaling | hsa04390 |
| PI3K-Akt signaling | hsa04151 |
| mTOR signaling | hsa04150 |
| VEGF signaling | hsa04370 |
| Longevity regulating | hsa04213, hsa04211 |
| Fc epsilon RI signaling | hsa04664 |
| Cytosolic DNA-sensing | hsa04623 |
| MAPK signaling | hsa04010 |
| Prolactin signaling | hsa04917 |
| ErbB signaling | hsa04012 |
| B cell receptor signaling | hsa04662 |
| Metabolic pathways | hsa01100 |
| TGF-beta signaling | hsa04350 |
| Adipocytokine signaling | hsa04920 |
| GnRH signaling | hsa04912 |
| TNF signaling | hsa04668 |
| Glucagon signaling | hsa04922 |
| PPAR signaling | hsa03320 |
| Toll-like receptor signaling | hsa04620 |
| Rap1 signaling | hsa04015 |
| T cell receptor signaling | hsa04660 |
| Ras signaling | hsa04014 |
| cAMP signaling | hsa04024 |
| Chemokine signaling | hsa04062 |
| Sphingolipid signaling | hsa04071 |
| Neurotrophin signaling | hsa04722 |
| Thyroid hormone signaling | hsa04919 |
| cGMP-PKG signaling | hsa04022 |
| Jak-STAT signaling | hsa04630 |
| Phospholipase D signaling | hsa04072 |
| Signaling pathways regulating pluripotency of stem cells | hsa04550 |

S4c. Pathways associated with stress response from WikiPathways database

| **Pathway** | **GO term** |
|---|---|
| MAPK Signaling | WP382 |
| EGFR1 Signaling | WP572 |
| Estrogen Receptor | WP2881 |
| Structural Pathway of Interleukin 1 (IL-1) | WP2637 |
| IL-1 signaling | WP195 |
| p38 MAPK Signaling | WP400/ WP350 |
| Toll-like Receptor Signaling | WP75/ WP1449 |
| Focal Adhesion-PI3K-Akt-mTOR-signaling | WP2841 |
| BDNF signaling | WP2380 |
| Delta-Notch Signaling | WP265 |
| TNF-alpha NF-kB Signaling | WP246 |
| EGF/EGFR Signaling | WP437 |
| NRF2 | WP2884 |
| Chemokine signaling | WP2292 |
| Androgen receptor signaling | WP138 |
| ATM Signaling | WP2516 |
| PPAR signaling | WP2316 |
| Hedgehog Signaling | WP116 |
| Mesodermal Commitment | WP2857 |
| Alpha6-Beta4 Integrin Signaling | WP488 |
| Inflammatory Response | WP458/ WP453 |
